# Associations Between Factors Affecting Itching and Quality of Life in Thai Patients with Psoriasis: A Cross-Sectional Study

**DOI:** 10.12688/f1000research.156703.2

**Published:** 2025-04-25

**Authors:** Phurichaya Teyateeti, Chanita Autchayawat, Wiriya Mahikul, Rithee Smithrithee

**Affiliations:** 1 Chulabhorn Hospital, Chulabhorn Royal Academy, Bangkok, 10210, Thailand; 2 Princess Srisavangavadhana College of Medicine, Chulabhorn Royal Academy, Bangkok, 10210, Thailand

**Keywords:** Itch; Pruritus; Psoriasis; Quality of life; Thailand

## Abstract

**Introduction:**

Psoriasis is a chronic skin disease affecting quality of life and causing pruritus. The factors influencing itch and its impact on the quality of life in Thai psoriasis patients are unknown. We aimed to identify these factors and their effect on quality of life.

**Methods:**

In this questionnaire-based cross-sectional study, we included patients with psoriasis who received treatment at Chulabhorn Hospital in Thailand from January 2019 to July 2021. Interviewer is the non-dermatologist practician. The patient’s information was collected, including demographic data, Itch Numeric Rating Scale (Itch NRS) score, factors affecting itch, and score on the Thai version of the Dermatology Life Quality Index (DLQI). We performed descriptive statistics and logistic regression analysis.

**Results:**

Of 100 participants, most (99%) experienced itching, with a moderate degree of pruritus (mean Itch NRS score 6.5 ± 2.6) and a moderate effect on quality of life (mean DLQI score 9.4 ± 6.2). Factors associated with itch aggravation were dry skin (p-value = 0.003) and heat and humidity (p-value = 0.042). The results of binary logistic regression revealed that factors associated with moderate-to-extremely large DLQI scores were itch intensity (no-to-mild vs. moderate-to-severe itch: odds ratio [OR] = 13.33; 95% confidence interval [CI] = 2.72–65.32, *p* < 0.001; and adjusted odds ratio [AOR] = 31.17; 95% CI = 4.55–213.36; *p* < 0.001.

**Conclusions:**

Our findings revealed that the quality of life among patients with psoriasis is their greatest concern. Eliminating the itch intensity that affects their quality of life is crucial but remains challenging in Thailand.

## Introduction

Psoriasis is among the diseases that most adversely affect quality of life. Psoriasis is a common, chronic immune-mediated inflammatory skin disease influenced by both genetic and environmental factors. The worldwide prevalence of psoriasis ranges from 0.91% to 8.5%, with people living in countries located closer to the equator tending to be affected by psoriasis less than those in more distant countries. ^1^ The burden of psoriasis not only involves the skin but also has the potential to cause other inflammatory conditions, such as arthritis, metabolic syndrome, cardiovascular diseases, inflammatory bowel diseases, and depression. ^2^ Therefore, a holistic approach to the management of patients with psoriasis is needed to improve overall outcomes and quality of life.

Psoriasis has not been regarded as being associated with pruritus until recently. Data have demonstrated that the prevalence of pruritus in patients with psoriasis ranges from 64% to 97%. ^3^ According to previous survey data, patients with psoriasis consider itching to be the most important factor responsible for disease severity, which differs from the opinion of dermatologists who only consider the location or the size of skin lesions. ^4^ A previous study conducted in Thailand extracted pruritus data from medical records of patients attending an outpatient psoriasis clinic between 2020 and 2021, revealing an overall prevalence of 81.2%. ^5^ The pathogenesis of pruritus in psoriasis remains unclear owing to complex interactions between the nervous, immune, neuroendocrine, and vascular systems. ^6, 7^ Thus, no promising treatment is available for pruritus in patients with psoriasis. ^8^ Moreover, factors identified to be associated with itching in patients with psoriasis differ across studies and may be affected by race and ethnicity, climate, and lifestyle. In this study, we aimed to investigate the association of pruritus with quality of life, the number of patients affected by itching, and factors associated with itching among Thai patients with psoriasis.

## Methods

### Study design & settings

The present research was a questionnaire-based cross-sectional study. We included patients diagnosed with psoriasis who received treatment at Chulabhorn Hospital in Thailand from January 2019 to July 2021. To estimate the sample size, we used the infinite population proportion formula: n = Z ^2^p(1−p)/d ^2^. Based on the assumption that 67% of patients reported a moderate to extremely large impact on their quality of life (DLQI > 6), ^9^ a 10% precision (d), a 95% confidence level, and accounting for a 10% non-response rate, the minimum calculated sample size was 95. The response rate was determined by dividing the number of respondents by the total number invited to the study. For example, 100 out of 125 individuals responded, yielding an 80% response rate. However, the total number of respondents still exceeds the sample size required to ensure sufficient statistical power for the study.

### Survey procedures

The inclusion criteria were patients over 18 years old without any cognitive or related problems, such as delirium or dementia. Each patient responded to the questionnaire by telephone (90%) or face-to-face (10%) with a non-dermatologist interviewer to avoid any bias. Alternatively, patients could choose to complete and return the questionnaire via postal mail. Patients were informed that they could stop the interview at any point and could also refuse to participate in the study without any effect on their treatment in the future. We collected information on patients’ demographic data, current treatment, Itch Numeric Rating Scale (Itch NRS) scores, ^10^ factors affecting itch, and scores on the Thai version of the Dermatology Life Quality Index (DLQI). ^11^

### Measures

For the assessment of itch intensity in this questionnaire-based study, we used the Itch NRS scale, a manual rating scale used to assess the intensity of pruritus, with scores ranging from 0 to 10. The Itch NRS was the instrument of choice because of our study design, its simplicity of assessment, and the high validity and high correlation with other manual rating scales, such as the visual analog scale (VAS) and verbal rating scale (VRS). ^10, 12, 13^ Quality of life was evaluated using the validated Thai version of DLQI was developed by Kulthanan K., et al., ^14^ a validated and widely used tool with a version available in Thailand. ^14^

### Statistical analysis

Data analysis using IBM SPSS software version 26 (IBM Corp., Armonk, NY, USA). Continuous variables are presented as mean with standard deviation. Categorical variables are summarized as frequency and percentage. To examine the association between different itchiness scales and factors, a chi-square test was performed, and p-values < 0.05 were considered statistically significant. Logistic regression analysis was conducted to assess the association between itching severity (dependent variable) and possible risk factors while controlling for potential confounding factors. Additionally, regression analysis was also conducted to assess the association between quality of life (dependent variable) and itching severity (independent variable) while controlling for potential confounding factors. Odds ratios (ORs) and adjusted odds ratios (AORs) with corresponding 95% confidence intervals (CIs) were estimated to quantify the association between itching severity and quality of life. Model fit statistics, such as the Hosmer–Lemeshow test and area under the receiver operating characteristic curve (AUC) were computed to assess the goodness-of-fit and predictive accuracy of the logistic regression model.

## Results

Overall, one hundred patients were enrolled in this study, all are Thai. Participants’ demographic data are shown in Table 1. Participants comprised 51 men and 49 women, with mean age 53.4 ± 15.2 years. Patients mean body mass index (BMI) was 25.0 ± 4.4 kg/m ^2^, which is classified as obese (BMI ≥25 kg/m ^2^ according to Asia Pacific guidelines). ^15^ Of the total, 45% of participants had a BMI of ≥ 25.0 kg/m ^2^, which is recognized as pre-obese and obese (BMI ≥ 25 kg/m ^2^). ^15^ Most patients were married (65%) and unemployed (37%), in the other word, pensioner. Among those who were employed, 31% were government employees. One-third of participants had no comorbidities; however, both hypertension and dyslipidemia were identified in 36 participants. Most patients were non-smokers and did not drink alcohol.

**Table 1.** Demographic data of patients (N = 100).

Variables	N = %
Sex	
Male	51
Female	49
Age (years)	
Mean ± SD	53.4 ± 15.2
≤ 35	17
36–59	44
≥ 60	39
BMI (kg/m ^2^)	
Mean ± SD	25.0 ± 4.4
< 18.5 (Underweight)	2
18.5–22.9 (Normal)	35
23–24.9 (Overweight)	18
≥ 25.0 (Obese)	45
Marital status	
Single	28
Married	65
Divorced	4
Widowed	3
Occupation	
Unemployed	37
Government employee	31
Non-government employee	32
Comorbidities	
Diabetes mellitus	21
Hypertension	36
Dyslipidemia	36
Cancer	10
Osteoarthritis	3
Coronary heart disease	4
Chronic kidney disease	5
Stroke	3
Fatty liver	3
Depression	1
No comorbidity	33
Smoking	
Yes	21
No	79
Alcohol consumption	
Yes	32 (31 cases, socially)
No	68
Treatment	
Topical medication	88
Systemic oral medication	17
Biologics	6
Antihistamines	35
Itch numeric rating scale	
Mean ± SD	6.5 ± 2.6
None (0)	1
Mild (1–3)	11
Moderate (4–6)	35
Severe (7–10)	53
DLQI score	
Mean ± SD	9.4 ± 6.2
No effect (0–1)	7
Small effect (2–5)	27
Moderate effect (6–10)	27
Very large and extremely large effect (11–30)	39

BMI, body mass index; SD, standard deviation; DLQI, Dermatology Life Quality Index.

The mean Itch NRS score was 6.5 ± 2.6 points. In our study, 88% of patients had moderate-to-severe itch, 11% reported mild itch, and only one patient had no itch. The mean DLQI score was 9.4 ± 6.2 points, which is classified as a moderate effect on quality of life. However, 39% of patients reported an extremely negative effect on their quality of life.

Among participants, 88% received topical treatment (topical corticosteroids, calcineurin inhibitors, or calcipotriene) and 17% received systemic oral medication (methotrexate, cyclosporine, or systemic retinoids). Only 6% received biologics as treatment. None of our patients had received phototherapy at the time of the interview. Moreover, 35% of patients were prescribed antihistamines for pruritus control.

Factors affecting pruritus are shown in Table 2. Factors leading to the aggravated itching among moderate-to-severe pruritus patients were dry skin (78.4%), poor sleep (65.9%), sweating (62.5%), stress (61.4%), and heat and humidity (59.1%). Factors associated with itch relief among moderate-to-severe pruritus patients were exercise (22.7%), followed by a cold environment and synthetic fabrics (11.4%, 9.1%, respectively).

**Table 2.** Factors affecting pruritus (N = 100).

Variables	Aggravated itching n (%)		Relieved itching n (%)		No effect n (%)		p-value
Itch NRS ^†^	0–3	4–10	0–3	4–10	0–3	4–10	
Stress	4 (33.3)	54 (61.4)	0 (0.0)	0 (0.0)	8 (66.7)	34 (38.6)	0.065
Dry skin	4 (33.3)	69 (78.4) *	0 (0.0)	0 (0.0)	8 (66.7)	19 (21.6)	0.003 *
Warm shower	2 (16.7)	22 (25.0)	0 (0.0)	0 (0.0)	10 (83.3)	66 (75.0)	0.725
Cold shower	0 (0.0)	2 (2.3)	1 (8.3)	6 (6.8)	11 (91.7)	80 (90.9)	0.762
Sweating	5 (41.7)	55 (62.5)	1 (8.3)	0 (0.0)	6 (50.0)	33 (37.5)	0.065
Heat and humidity	4 (33.3)	52 (59.1) *	1 (8.3)	0 (0.0)	7 (58.3)	36 (40.9)	0.042 *
Cold environment	1 (8.3)	33 (37.5)	1 (8.3)	10 (11.4)	10 (83.3)	45 (51.1)	0.064
Poor sleep	5 (41.7)	58 (65.9)	0 (0.0)	1 (1.1)	7 (58.3)	29 (33.0)	0.224
Exercise	1 (8.3)	8 (9.1)	4 (33.3)	20 (22.7)	7 (58.3)	60 (68.2)	0.736
Natural fabrics	0 (0.0)	10 (11.4)	1 (8.3)	5 (5.7)	11 (91.7)	73 (83.0)	0.251
Synthetic fabrics	1 (8.3)	8 (9.1)	0 (0.0)	8 (9.1)	11 (91.7)	72 (81.8)	0.338

* *p* < 0.05, chi-square test.

^†^ Itch Numerical Rating Scale (NRS) scores were divided into groups with no-to-mild (score 0–3) and moderate-to-severe (score 4–10) itch.

A chi-square test indicated significant differences in itchiness scales between the group with no-to-mild itch and the group with moderate-to-severe itch for two factors: dry skin ( *p*-value = 0.003) and heat and humidity ( *p*-value = 0.042). Other factors, including stress, warm or cold shower, sweating, cold environment, poor sleep, exercise, natural fabrics, and synthetic fabrics, were not associated with either aggravating or relieving itching.

The results of binary logistic regression revealed no significant association between itching and sex, age, BMI, marital status, occupation, comorbidities, smoking, or drinking alcohol. However, the results of binary logistic regression revealed that factors related to a moderate-to-extremely large DLQI were itch intensity (no-to-mild vs. moderate-to-severe itch: OR = 13.33; 95% CI = 2.72–65.32, *p* < 0.001 and AOR = 31.17; 95% CI = 4.55–213.36, *p* < 0.001) as shown in the Table 3.

**Table 3.** Factors related to DLQI.

Variables	DLQI, n (%)		OR (95% CI)		*p*-value
	No to small (≤ 5 score)	Moderate to large (>5 score)	Crude	Adjusted	
Sex					
Male	13 (25.5)	38 (74.5)	1.00	1.00	
Female	21 (42.9)	28 (57.1)	0.46 (0.19,1.06)	0.42 (0.11,1.58)	0.198
Age (years)					
≤ 35	5 (29.4)	12 (70.6)	1.00	1.00	
36–59	13 (29.5)	31 (70.5)	0.99 (0.29,3.39)	0.95 (0.16,5.56)	0.951
≥ 60	16 (41.0)	23 (59.0)	0.59 (0.18,2.04)	0.53 (0.07,4.01)	0.543
BMI (kg/m ^2^)					
< 25.0	19 (34.5)	36 (65.5)	1.00	1.00	
≥ 25.0	15 (33.3)	30 (66.7)	1.06 (0.46,2.43)	1.18 (0.42,3.32)	0.762
Marital status					
Single	8 (28.6)	20 (71.4)	1.00	1.00	
Married or Widowed	26 (36.1)	46 (63.9)	0.71 (0.27,1.83)	0.60 (0.15,2.36)	0.468
Occupation					
Unemployed	15 (40.5)	22 (59.5)	1.00	1.00	
Government employee	10 (32.3)	21 (67.7)	1.43 (0.53,3.89)	1.09 (0.29,4.11)	0.899
Non-government employee	9 (28.1)	23 (71.9)	1.74 (0.63,4.79)	1.92 (0.46,7.96)	0.371
Comorbidities					
No	10 (30.3)	23 (69.7)	1.00	1.00	
Yes	24 (35.8)	43 (64.2)	0.78 (0.32,1.91)	0.77 (0.21,2.76)	0.685
Smoking					
No	29 (36.7)	50 (63.3)	1.00	1.00	
Yes	5 (23.8)	16 (76.2)	1.86 (0.62,5.59)	2.80 (0.49,15.84)	0.244
Alcohol consumption					
No	25 (36.8)	43 (63.2)	1.00	1.00	
Yes	9 (28.1)	23 (71.9)	1.49 (0.59,3.71)	1.05 (0.26,4.24)	0.950
Topical medication					
No	6 (50.0)	6 (50.0)	1.00	1.00	
Yes	28 (31.8)	60 (68.2)	2.14 (0.63,7.24)	0.21 (0.01,15.72)	0.479
Systemic oral medication					
No	28 (33.7)	55 (66.3)	1.00	1.00	
Yes	6 (35.3)	11 (64.7)	0.93 (0.31,2.79)	1.88 (0.42, 8.40)	0.411
Biologics					
No	33 (35.1)	61 (64.9)	1.00	1.00	
Yes	1 (16.7)	5 (83.3)	2.71 (0.30,24.13)	2.09 (0.14,31.65)	0.595
Antihistamines					
No	24 (36.9)	41 (63.1)	1.00	1.00	
Yes	10 (28.6)	25 (71.4)	1.46 (0.60,3.56)	1.32 (0.42,4.12)	0.639
Itch numeric rating scale					
No to mild	10 (83.3)	2 (16.7)	1.00	1.00	
Moderate to severe	24 (27.3)	64 (72.7)	13.33 (2.72,65.32)	31.17 (4.55,213.36)	<0.001 *

* *p* < 0.05.

## Discussion

Psoriasis is a common, chronic immune-mediated inflammatory skin disease that affects not only the skin but can also cause other inflammatory conditions. Unlike the concerns of dermatologists, itching is one of the most concerning symptoms among patients with psoriasis. ^4^ Itching in psoriasis may involve any area of the body, with the lesional skin affected more than non-lesional skin, usually spare face and neck area, or it can be generalized in some patients. ^16, 17^ Pruritus tends to appear in the evening and at night. ^18^ The common characteristics of pruritus described by patients include stinging, tickling, and crawling sensations. Moreover, a number of patients report pain and heat as accompanying sensations. ^16^

Our study results revealed itching in 99% of patients or almost all the patients, which was surprisingly high compared to previous data of 64%–97%. ^3^^,^^5^ Among all patients, 88% had moderate-to-severe itching. The only patient without pruritus was a patient with nail psoriasis, which is considerably less likely to involve itching. ^19^ Our data (mean Itch NRS score 6.5 ± 2.6) were correlated with previous data reported by Yosipovitch et al, Reich et al, Amatya et al, and Damiani et al, in that most patients with psoriasis had pruritus to a moderate degree (mean VAS of 6.4 ± 2.5 [the worst], 5.2 ± 2.4, 5.2 ± 2.6, and 4.6 ± 3.2, respectively). ^16, 18, 20, 21^ The NRS and VAS were validated to have a high correlation. ^12^ Emphasizing that itching is the major factor effecting quality of life in psoriasis patients.

Approximately one-quarter of our patients received systemic treatment, either oral systemic or biologic drugs; the remainder (77% of patients) had limited disease severity or less than 10% body surface area involvement. These data may imply that even in patients with mildly severe psoriasis, regardless of the psoriasis area and severity index (PASI), itching can still occur. Similarly, other studies have reported no significant correlation between itch intensity and PASI score. ^8, 18, 22– 25^ Moreover, the mismatch of an improvement in PASI without an improvement in itching indicates that PASI cannot completely represent itching in patients with psoriasis, and not represent successful treatment for psoriasis patients. ^19^ This emphasizes the role of itch control in these patients, not only in those with higher PASI but also in those with a milder form of psoriasis. However, some authors have reported that the intensity of pruritus is correlated with the severity of psoriasis. ^26, 27^ The disparity in these data may be affected by the differences in study populations with respect to the severity of disease, treatment received, race and ethnicity, and local climate.

The mean DLQI score of patients with psoriasis in our study was 9.4 ± 6.2, which is classified as representing a moderate effect of itching on quality of life. Similarly, most other studies have reported a moderate-to-very large effect on QOL. Several studies reporting a moderate effect of psoriasis on quality of life were conducted in Iran, ^28^ Taiwan, ^9^ and Malaysia, ^29^ with a mean DLQI of 8, 9.2 ± 6.3, and 6 (median, interquartile range 4.0), respectively. While studies in Poland, ^30^ Greece, ^31^ Korea, ^32^ and China ^33^ have reported an exceptionally large effect on quality of life, with a mean DLQI of 17.8 ± 5.9, 12.6 ± 4.9, 12.4 ± 7.6, and 13.3, respectively. In our study, 66% of patients reported a moderate-to-extremely large effect, according to DLQI score. Moreover, our data indicated that itch intensity was significantly associated with patients’ DLQI, similarly to those of other studies. ^18, 22, 24, 34^ Many studies also indicated that itching was not only associated with the DLQI of patients with psoriasis but was also correlated with feelings of stigmatization, stress within the month before a psoriasis outbreak, anxiety, sleep disturbance, fatigue, and various depressive symptoms. ^22, 24, 34^ Female patients reported a significantly negative impact on quality of life and are more likely to have lower self-esteem than male patients. ^31, 32^ Younger age has been identified as an independent risk factor for psychological complications and severely impaired quality of life in patients with psoriasis. ^35^ Moreover, patients with psoriasis who have lesions on both sensitive and non-sensitive areas (genitals, scalp, and face) had significantly increased DLQI scores compared with patients who had lesions on non-sensitive body areas only. ^34^ Furthermore, regardless of the cause, the severity of pruritus also affects the patient’s quality of life. ^36^

For the factors that aggravating itching, dry skin (73%), poor sleep (63%), sweating (60%), stress (58%), and heat and humidity (56%) were revealed. Concordance to previous studies, mostly reported as factors aggravating itching in previous studies were stress, sweating, being sick or unwell, a hot ambient temperature, dryness or dry skin, and the winter season. ^16, 18, 19, 25, 37^ A study in Italy uniquely reported that significant aggravating factors included hot water, exercise, large meals, bad mood, the supine position, and contact with clothes. ^37^ This study found that itch-relieving factors included exercise (24%), a cold environment (11%), synthetic fabric (8%), and a cold shower (7%). Those factors most commonly reported as itch relievers from previous studies included sleep, cold water, and the sun. ^16, 18, 37^ However, our data indicated only two factors to be significantly associated with itch aggravation: dry skin ( *p*-value = 0.003) and heat and humidity ( *p*-value = 0.042). A positive correlation between pruritus intensity and stress was reported by Amatya et al. (r = 0.8, p < 0.05) and Reich et al. (p = 0.015). ^16, 21^ Heat or warm stimuli can provoke itch via transient receptor potential vanilloid subtype 1 (TRPV1) and calcitonin gene-related peptide (CGRP), both of which are involved in the pathogenesis of itching with psoriasis. ^7, 38^ In summary, studies in countries with subtropical climates, such as Thailand and Singapore, report hot temperatures and sweating as itch aggravators and cold water and cold temperatures as itch relievers. However, a study from Sweden in Northern Europe described sun as an itch reliever. This data indicate that climate also influences itch symptoms. Patients with psoriasis in countries with different climates may have different factors that aggravate or relieve itching symptoms. The hypothesis may be, in a warm and moist weather zone, the cold environment relieving itching from every daily life sweating, and warm. In contrary, from the patients from cold and dry weather zone, the sweating and high humidity may alleviate the symptoms. The changing of the surrounding environment might be the key to improving QOL in psoriatic patients, as in climatotherapy.

There was no statistically significant correlation between itching and sex, age, BMI, marital status, occupation, comorbidities, smoking, or drinking alcohol in this study. A large cohort study from Italy with 10,802 participants also reported no statistical significance between itching and age, BMI, marital status, smoking, or drinking alcohol. ^20^ Nevertheless, female sex was significantly associated with itch intensity, with both sexes having a mean VAS score indicating moderate severity. ^20^ Similarly, Amatya et al. demonstrated that women more often reported itching than men (OR, 3.8; 95% CI, 2–15; *p* < 0.05). ^16^ Conversely, some studies found no correlation between sex and itching. ^18, 25, 26^ When the disease is not active, female patients with psoriasis had a significantly lower VAS score than male patients. ^18^ Variations in study populations, number of participants, and severity of disease in each study may affect variation in the correlations between itching and factors influencing itch intensity.

Owing to the complex pathogenesis of pruritus in psoriasis, no promising treatment is available for pruritus in patients with psoriasis. In our study, 35% of patients were prescribed antihistamines for itch control. However, there was no significant difference in the mean plasma concentration of histamine between patients with psoriasis and controls, nor between patients who had pruritic psoriasis, those with non-pruritic psoriasis, and controls. ^39, 40^ Thus, histamine may not be a key mediator in psoriasis-associated pruritus. ^41^ Nonetheless, most data indicate that antihistamines have no or only short-term effects. ^16, 18, 42, 43^ Topical treatments were used by 88% of our participants. According to some studies, topical corticosteroids have more short-term effects than long-term benefits or have no antipruritic effect at all. ^16, 18^

A fixed-dose combination of betamethasone dipropionate and calcipotriene has demonstrated significant itch relief, with rapid onset. ^44, 45^ Phototherapy has been reported to have variable outcomes. Yosipovitch et al. found that 65% of patients who received UVB phototherapy reported no antipruritic effect, and the remaining patients reported more short-term benefits than long-term ones. ^18^ Additionally, Amatya reported an equal percentage of benefit in both the short and long term. ^16^ However, Narbutt et al. found a significant reduction in pruritus scores after 10 irradiations, and two-thirds of patients also reported no itch symptoms after irradiation. ^46^ Methotrexate and systemic retinoids had no effect on itch reduction. ^16, 18^ In contrast, cyclosporine was reported to significantly relieve itching in patients with psoriasis. ^47^ Biologics can lead to itch improvement. ^47^ Of all biologics, anti-interleukin 17 showed the most significant magnitude in terms of itch reduction among patients with psoriasis. ^48^ However, we did not evaluate itch responses according to each treatment modality in our study.

### Limitations

Limitations in our study include the specific patients with small sample size, population bias with mild severity of disease, and no available PASI score. This study might not represent all the psoriasis patients due to our institute taking care only of the patient with health care benefit. On the other hands, the optimized treatment may unrevealed the effect of itching on the well-treated psoriasis patients. However, itching still affected the quality of life of our patients. Additionally, there are many more factors that influence itching and quality of life in patients with psoriasis beyond our scope, and that vary by country. Other causes of pruritus, such as kidney, liver, and hematologic diseases, may affect these data. However, the presence of systemic disease has shown no significant effect on pruritus in patients with psoriasis. ^49^ Increasing the study population might improve the significance of the results. Our study relied on self-reported factors and itch outcomes, which should not be interpreted as confirmed clinical measurements or diagnoses. Lastly, this study used a telephone-based cross-sectional survey method during the COVID-19 pandemic to minimize the risk of transmission. As a result, causal relationships could not be established, and the sample was limited to individuals with telephone access. Therefore, the findings may not fully represent the entire population.

## Conclusions

Itching is a problem for most patients with psoriasis and optimizing itch control remains challenging. Our study found that most participants had a moderate-to-severe degree of pruritus, and itch intensity also had a statistically significant association with DLQI score. Factors identified as being significantly associated with itch aggravation were dry skin as well as heat and humidity. Identifying all the possible factors aggravating psoriasis is the most important to improve QoL. Pruritus control will benefit patients by both preventing the Koebner phenomenon and improving their QoL. A comprehensive approach is needed to provide the best outcome for all patients with psoriasis.

## Ethics and consent

Prior to the commencement of this study, ethical approval was granted by the Human Research Ethics Committee of Chulabhorn Royal Academy, Thailand (EC 042/2564) on 15 ^th^ October 2021 for 6 months, and it conformed to the ethics guidelines of the Declaration of Helsinki. All procedures were performed in accordance with relevant guidelines and regulations. Additionally, the study received approval from the general community. All participants in the study provided signed informed consent before completing the questionnaires.

## Data Availability

Associations between factors affecting itching and quality of life in Thai patients with psoriasis: A cross-sectional study Figshare: https://doi.org/10.6084/m9.figshare.27042550 ^50^ This project contains the following underlying data:
• Data-Excel.xlsx Data are available under the terms of the Creative Commons Attribution 4.0 International license (CC-BY 4.0). Associations between factors affecting itching and quality of life in Thai patients with psoriasis: A cross-sectional study Figshare: https://doi.org/10.6084/m9.figshare.27181140 ^51^ This project contains the following extended data:
• Questionnaire.docx Data are available under the terms of the Creative Commons Attribution 4.0 International license (CC-BY 4.0).
