# Supplementary material for: Associations Between Factors Affecting Itching and Quality of Life in Thai Patients with Psoriasis: A Cross-Sectional Study
Source: F1000Res. 2025 Apr 25;13:1242. Originally published 2024 Oct 17. [Version 2] doi: 10.12688/f1000research.156703.2 (PMC12103706; doi:10.12688/f1000research.156703.2)
Supplement: Peer review history

# Reviewer report: Reviewer response for version 2

**DOI:** 10.5256/f1000research.181039.r381203

**Published:** 2025-05-24

**Authors:** Adam Reich

**Affiliations:** 1 Department of Dermatology, University of Rzeszów, Rzeszów, Poland

The authors performed a study among Thai patients with psoriasis on the prevalence and intensity of itching. I found the study interesting, relevant, and well-performed. The results are important, as they document a high prevalence of itch in psoriasis patients (99%) and a significant impact of pruritus on quality of life. Regarding data presentation, I would suggest reconsidering the following statement: " (...) most (99%) experienced itching, with a moderate degree of pruritus (mean Itch NRS score 6.5 ± 2.6) and a moderate effect on quality of life (mean DLQI score 9.4 ± 6.2). Please note that these are mean values. However, about 50% of patients demonstrate DLQI scoring >10, which indicates a very large or extremely large effect on QoL. The same applies to NRS, in which a substantial proportion of patients had NRS scoring >7, which indicates high pruritus intensity. 

 Please modify reference 21 as follows:

 Reich A, Szepietowski JC, Wisnicka B, *et al.*: Does Stress Influence Itching in Psoriatic Patients? *Dermatology and Psychosomatics/Dermatologie und Psychosomatik.* 2003 09/01; **4**: 151–155

Is the work clearly and accurately presented and does it cite the current literature?

Yes

If applicable, is the statistical analysis and its interpretation appropriate?

Yes

Are all the source data underlying the results available to ensure full reproducibility?

Yes

Is the study design appropriate and is the work technically sound?

Yes

Are the conclusions drawn adequately supported by the results?

Yes

Are sufficient details of methods and analysis provided to allow replication by others?

Yes

Reviewer Expertise:

Pruritus, psychodermatology, psoriasis

I confirm that I have read this submission and believe that I have an appropriate level of expertise to confirm that it is of an acceptable scientific standard.

# Reviewer report: Reviewer response for version 2

**DOI:** 10.5256/f1000research.181039.r380765

**Published:** 2025-05-14

**Authors:** Narumol Silpa-archa

**Affiliations:** 1 Dermatology Faculty of Medicine Siriraj Hospital, Mahidol University, Bangkok, Mahidol University,, Thailand

Following the first revisions, the manuscript becomes more knowledgeable and well-written. The authors responded well, based on the comments.

Is the work clearly and accurately presented and does it cite the current literature?

Partly

If applicable, is the statistical analysis and its interpretation appropriate?

I cannot comment. A qualified statistician is required.

Are all the source data underlying the results available to ensure full reproducibility?

Partly

Is the study design appropriate and is the work technically sound?

Yes

Are the conclusions drawn adequately supported by the results?

Yes

Are sufficient details of methods and analysis provided to allow replication by others?

Partly

Reviewer Expertise:

Photodermatology

I confirm that I have read this submission and believe that I have an appropriate level of expertise to confirm that it is of an acceptable scientific standard.

# Reviewer report: Reviewer response for version 1

**DOI:** 10.5256/f1000research.172051.r371560

**Published:** 2025-04-22

**Authors:** Narumol Silpa-archa

**Affiliations:** 1 Dermatology Faculty of Medicine Siriraj Hospital, Mahidol University, Bangkok, Mahidol University,, Thailand

The authors did an interesting cross-sectional study on itch and DLQI in Thai psoriasis. These are questions and comments.

 1. How many patients were interviewed by phone and how many were questioned on-site? Please provide additional information.

 2. Are there any data on psoriasis severity, BSA, PGA, or PASI from onsite patients? Please clarify, as this is one of the major causes of itching in psoriasis.

 3. The authors determined that dry skin, heat, and humidity were key causes of itching. Please provide further information about dry skin (any measurements) and season affect (winter or summer). These can provide additional information to the reader.

 4. Which locations are most affected by psoriasis itching, according to this study?

 5. To increase the value of the manuscript, it should be examined by a native English speaker.

 6. The author can add a published study on itch in Thai psoriasis patients, and add comparison in discussion part. 

 Please see [Ref 1]

Is the work clearly and accurately presented and does it cite the current literature?

Partly

If applicable, is the statistical analysis and its interpretation appropriate?

I cannot comment. A qualified statistician is required.

Are all the source data underlying the results available to ensure full reproducibility?

Partly

Is the study design appropriate and is the work technically sound?

Yes

Are the conclusions drawn adequately supported by the results?

Yes

Are sufficient details of methods and analysis provided to allow replication by others?

Partly

Reviewer Expertise:

Photodermatology

I confirm that I have read this submission and believe that I have an appropriate level of expertise to confirm that it is of an acceptable scientific standard, however I have significant reservations, as outlined above.

## References

[1] : Prevalence and clinical characteristics of pruritus, and the factors significantly associated with high pruritic intensity in patients with psoriasis: a cross-sectional study. *Ann Med Surg (Lond)* .2023;85(7) : 10.1097/MS9.0000000000000948 3396-3402 10.1097/MS9.0000000000000948 37427235 PMC10328595

# Response

**Published:** 2025-04-22

**Authors:** Wiriya Mahikul

**Affiliations:** Faculty of Medicine, Chulabhorn Royal Academy, Bangkok, Bangkok, Thailand

**Reviewer 2**

 Narumol Silpa-archa, Dermatology Faculty of Medicine Siriraj Hospital, Mahidol University, Bangkok, Mahidol University, Thailand

 Approved With Reservations

 info_outline

 The authors did an interesting cross-sectional study on itch and DLQI in Thai psoriasis. These are questions and comments.

 1. How many patients were interviewed by phone and how many were questioned on-site? Please provide additional information.

 Thank you for your comments. We agree with the reviewer. Upon rechecking the data, we found that approximately 90% of participants were assessed via telephone interviews for itchiness and quality of life due to the survey being conducted during the COVID-19 pandemic, while only 10% participated in face-to-face interviews. We have clarified this in the Survey Procedures and Limitations sections, as follows:

 Survey procedures: ' Each patient responded to the questionnaire by telephone (90%) or face-to-face (10%) with a non-dermatologist interviewer to avoid any bias.' - line 78-79

 Limitations sections: 'Lastly, this study used a telephone-based cross-sectional survey method during the COVID-19 pandemic to minimize the risk of transmission. As a result, causal relationships could not be established, and the sample was limited to individuals with telephone access. Therefore, the findings may not fully represent the entire population'- line 279-282

 2. Are there any data on psoriasis severity, BSA, PGA, or PASI from onsite patients? Please clarify, as this is one of the major causes of itching in psoriasis.

 Thank you for your comments. However, we did not have any data on psoriasis severity, BSA, PGA, or PASI from onsite patients.

 We added this in the limitation as following.

 'Limitations in our study include the specific patients with small sample size, population bias with mild severity of disease, and no available PASI score.' - line 281

 3. The authors determined that dry skin, heat, and humidity were key causes of itching. Please provide further information about dry skin (any measurements) and season affect (winter or summer). These can provide additional information to the reader.

 The assessment was based on patient self-reports regarding the effects of dry skin, heat and humidity, and seasonal factors (e.g., cold environments) on their itch symptoms. We asked patients how these factors influenced their itch—whether symptoms worsened, improved, or remained unchanged. As this information is self-reported, it may not be as accurate as standardized measurements; however, this limitation has been acknowledged in the manuscript as follow;

 'Our study relied on self-reported factors and itch outcomes, which should not be interpreted as confirmed clinical measurements or diagnoses' - line 281-282

 4. Which locations are most affected by psoriasis itching, according to this study?

 We did not have data on the locations most affected by psoriasis-related itching because we did not ask participants to specify the location of their itch. Instead, we used the Itch Numeric Rating Scale (Itch NRS), which is typically used to assess overall or general itch intensity, often referring to whole-body itch.

 5. To increase the value of the manuscript, it should be examined by a native English speaker.

 Thank you for your comments. We have had some sentences revised by a native English speaker. We thank Analisa Avila, MPH, ELS, of Edanz (www.edanz.com/ac) for editing a draft of this manuscript.

 6. The author can add a published study on itch in Thai psoriasis patients, and add comparison in discussion part. 

 Please see [Ref 1] : 1. Junsuwan N, Likittanasombat S, Chularojanamontri L, Chaiyabutr C, et al.: Prevalence and clinical characteristics of pruritus, and the factors significantly associated with high pruritic intensity in patients with psoriasis: a cross-sectional study. *Ann Med Surg (Lond)*. 2023; **85** (7): 3396-3402 PubMed Abstract | Publisher Full Text

 Thank you for your advice. We have added this reference in the Introduction and included a comparison in the Discussion section as follows:

 In introduction as 'A previous study conducted in Thailand extracted pruritus data from medical records of patients attending an outpatient psoriasis clinic between 2020 and 2021, revealing an overall prevalence of 81.2% ^3^' - line 57-59

 In discussion as ' Our study results revealed itching in 99% of patients or almost all the patients, which was surprisingly high compared to previous data of 64%–97% ^3, 4^^'^. - line 184

**Reference**

 1.          Lin TY, See LC, Shen YM, Liang CY, Chang HN, Lin YK. Quality of life in patients with psoriasis in northern Taiwan. Chang Gung Med J. 2011 Mar-Apr;34(2):186-96. PubMed PMID: 21539761. Epub 2011/05/05.

 2.          Consultation WHOE. Appropriate body-mass index for Asian populations and its implications for policy and intervention strategies. Lancet. 2004 Jan 10;363(9403):157-63. PubMed PMID: 14726171. Epub 2004/01/17.

 3.          Junsuwan N, Likittanasombat S, Chularojanamontri L, Chaiyabutr C, Wongpraparut C, Silpa-Archa N. Prevalence and clinical characteristics of pruritus, and the factors significantly associated with high pruritic intensity in patients with psoriasis: a cross-sectional study. Ann Med Surg (Lond). 2023 Jul;85(7):3396-402. PubMed PMID: 37427235. Pubmed Central PMCID: PMC10328595. Epub 2023/07/10. eng.

 4.          Tsianakas A, Mrowietz U. [Pruritus in psoriasis : Profile and therapy]. Hautarzt. 2016 Aug;67(8):601-5. PubMed PMID: 27376751. Epub 2016/07/05. Pruritus bei Psoriasis : Profil und Therapie.

# Reviewer report: Reviewer response for version 1

**DOI:** 10.5256/f1000research.172051.r364699

**Published:** 2025-02-28

**Authors:** Napatra Tovanabutra

**Affiliations:** 1 Chiang Mai University, Chiang Mai, Chiang Mai, Thailand

Overall, this is a well-written manuscript which evaluates the association between factors affecting itch and quality of life in Thai patient with psoriasis. The result of the analysis seems accurate. The following are minor comments which need to be addressed and revised.

- Due to this study allowing the patient response to the questionnaire by telephone, it would be good if the authors can report the number of patients who response by the different methods and whether the difference of the results between these two methods.
- Authors should demonstrate the sample-size calculations.
- According to the study involved Thai patients, Asian-Pacific BMI should be used instead of WHO BMI.

Is the work clearly and accurately presented and does it cite the current literature?

Yes

If applicable, is the statistical analysis and its interpretation appropriate?

I cannot comment. A qualified statistician is required.

Are all the source data underlying the results available to ensure full reproducibility?

Yes

Is the study design appropriate and is the work technically sound?

Yes

Are the conclusions drawn adequately supported by the results?

Yes

Are sufficient details of methods and analysis provided to allow replication by others?

Yes

Reviewer Expertise:

Dermatologist

I confirm that I have read this submission and believe that I have an appropriate level of expertise to confirm that it is of an acceptable scientific standard, however I have significant reservations, as outlined above.

# Response

**Published:** 2025-03-03

**Authors:** Wiriya Mahikul

**Affiliations:** Faculty of Medicine, Chulabhorn Royal Academy, Bangkok, Bangkok, Thailand

**Reviewer 1**

 Napatra Tovanabutra, Chiang Mai University, Chiang Mai, Chiang Mai, Thailand

 Approved With Reservations

 info_outline

**Overall, this is a well-written manuscript which evaluates the association between factors affecting itch and quality of life in Thai patient with psoriasis. The result of the analysis seems accurate. The following are minor comments which need to be addressed and revised.**

 Thank you for the positive reviews on our manuscript and for the reviewers’ comments on how to improve the publication.

**Due to this study allowing the patient response to the questionnaire by telephone, it would be good if the authors can report the number of patients who response by the different methods and whether the difference of the results between these two methods.**

 Thank you for your comments. We agree with the reviewer. Upon rechecking the data, we found that approximately 90% of participants were assessed via telephone interviews for itchiness and quality of life due to the survey being conducted during the COVID-19 pandemic, while only 10% participated in face-to-face interviews. This small proportion of face-to-face interviews was insufficient to perform a subgroup analysis comparing the two methods. We have clarified this in the Survey Procedures and Limitations sections, as follows:

 Survey procedures: ' Each patient responded to the questionnaire by telephone (90%) or face-to-face (10%) with a non-dermatologist interviewer to avoid any bias.' - line 78-79

 Limitation: 'Lastly, this study used a telephone-based cross-sectional survey method during the COVID-19 pandemic to minimize the risk of transmission. As a result, causal relationships could not be established, and the sample was limited to individuals with telephone access. Therefore, the findings may not fully represent the entire population.' - line 286-289

**Authors should demonstrate the sample-size calculations.**

 Thank you for your comments. We have provided the sample size calculation as follows: 'To estimate the sample size, we used the infinite population proportion formula: n = Z²p(1-p)/d². Based on the assumption that 67% of patients reported a moderate to extremely large impact on their quality of life (DLQI > 6) ^1^, a 10% precision (d), a 95% confidence level, and accounting for a 10% non-response rate, the minimum calculated sample size was 95. The response rate was determined by dividing the number of respondents by the total number invited to the study. For example, 100 out of 125 individuals responded, yielding an 80% response rate. However, the total number of respondents still exceeds the sample size required to ensure sufficient statistical power for the study.' - line 68-75

**According to the study involved Thai patients, Asian-Pacific BMI should be used instead of WHO BMI.**

 We agree with the reviewer. Therefore, we changed the BMI classification from the WHO BMI to the Asian-Pacific BMI, as the study involved Thai patients, as follows: 'Patients mean body mass index (BMI) was 25.0 ± 4.4 kg/m ^2^, which is classified as obese (BMI ≥25 kg/m ^2^ according to Asia Pacific guidelines) ^2^. Of the total, 45% of participants had a BMI of ≥ 25.0 kg/m ^2^, which is recognized as obese (BMI ≥ 25 kg/m ^2^) ^2^.' - line 117-119 and Table 1

**Table 1.** Demographic data of patients (N = 100)

 Variables                                N = %

 BMI (kg/m ^2^)

   Mean ± SD                          25.0 ± 4.4

    < 18.5 (Underweight)          2

    18.5–22.9 (Normal)            35

    23–24.9 (Overweight)         18

    ≥ 25.0 (Obese)                    45

 However, this change does not affect our main results, as there is still no significant difference between BMI and DLQI.

 Is the work clearly and accurately presented and does it cite the current literature?

 Yes

 Is the study design appropriate and is the work technically sound?

 Yes

 Are sufficient details of methods and analysis provided to allow replication by others?

 Yes

 If applicable, is the statistical analysis and its interpretation appropriate?

 I cannot comment. A qualified statistician is required.

 Are all the source data underlying the results available to ensure full reproducibility?

 Yes

 Are the conclusions drawn adequately supported by the results?

 Yes

 Competing Interests

 No competing interests were disclosed.

 Reviewer Expertise

 Dermatologist

 I confirm that I have read this submission and believe that I have an appropriate level of expertise to confirm that it is of an acceptable scientific standard, however I have significant reservations, as outlined above.

**Reference**

 1.         Lin TY, See LC, Shen YM, Liang CY, Chang HN, Lin YK. Quality of life in patients with psoriasis in northern Taiwan. Chang Gung Med J. 2011 Mar-Apr;34(2):186-96. PubMed PMID: 21539761. Epub 2011/05/05.

 2.         Consultation WHOE. Appropriate body-mass index for Asian populations and its implications for policy and intervention strategies. Lancet. 2004 Jan 10;363(9403):157-63. PubMed PMID: 14726171. Epub 2004/01/17.
